# Emergency department diagnosis of a quadriceps intramuscular loculated abscess/pyomyositis using dynamic compression bedside ultrasonography

**DOI:** 10.1186/2036-7902-5-3

**Published:** 2013-02-13

**Authors:** Aleksandr Tichter, David C Riley

**Affiliations:** 1 Emergency Medicine Department, Columbia University Medical Center, New York, NY, USA

**Keywords:** Ultrasound, Quadriceps intramuscular loculated abscess, Pyomyositis, Emergency department

## Abstract

**Introduction:**

A 73-year-old man with a past medical history of myelodysplastic syndrome and recent chemotherapy presented to the emergency department with a 1-week history of progressively increasing left thigh pain and swelling. His physical examination revealed left anterolateral diffuse thigh swelling with no erythema or warmth to palpation. The anterolateral quadriceps was markedly tender to palpation. Emergency department bedside dynamic compression ultrasonography that was performed on the left anterolateral thigh revealed a quadriceps intramuscular abscess with loculated yet movable pus.

**Conclusion:**

Bedside dynamic compression ultrasonography can assist the emergency or critical care physician in the diagnosis of quadriceps intramuscular abscess or pyomyositis.

## Background

Pyomyositis is a suppurative infection of the skeletal muscle, usually involving the proximal lower extremities, which typically involves single isolated muscles such as the quadriceps muscle and, if left untreated, often evolves into an organized collection or abscess which can progress to sepsis and septic shock if untreated [1]. Traditionally considered a disease endemic to warmer climates, therefore earning the designation ‘tropical pyomyositis’, it has recently become more prevalent in temperate regions, usually arising in predisposing immunocompromised patients with conditions such as diabetes, bone marrow malignancies, and human immunodeficiency virus (HIV) infection as 21% of patients with pyomyositis are HIV positive [2]. Emergency physicians using point-of-care bedside ultrasonography to rapidly diagnose an intramuscular abscess or pyomyositis can expedite rapid orthopedic surgical consultation after immediate emergency department (ED) intravenous broad-spectrum antibiotic administration.

## Case presentation

**Figure 1:**
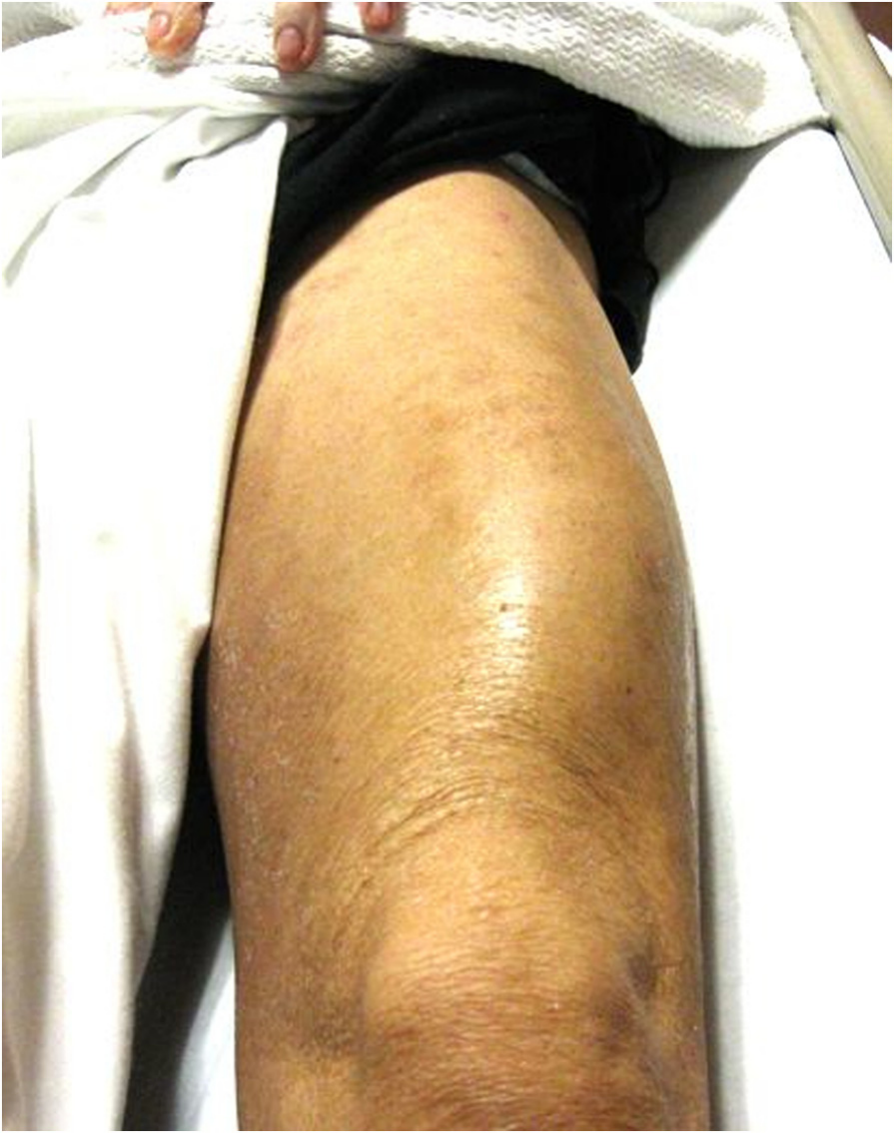
Swelling of the left quadriceps, intramuscular abscess, and pyomyositis.

A 73-year-old man with a past medical history of myelodysplastic syndrome and recent chemotherapy presented to the emergency department with a 1-week history of progressively increasing left thigh pain and swelling that has prevented him from walking for several days. He denied any trauma, shortness of breath, chest pain, or fever or chills. His ED vital signs were as follows: temperature, 100.3°F; blood pressure, 110/80 mmHg; respiratory rate, 16 beats per minute (bpm); room air oxygen saturation, 98%; and heart rate, 112 bpm. His physical examination revealed left anterolateral diffuse thigh swelling (Figure 1) with no erythema or warmth to palpation. The anterolateral quadriceps was markedly tender to palpation. There was no popliteal tenderness and no pretibial edema. Femur X-ray showed soft tissue prominence with no subcutaneous air. Laboratory studies revealed a white blood cell count of 3.5 (normal 3.54 to 9.06 × 10^9^/l) with 62% neutrophils (normal 40% to 70%).

**Figure 2:**
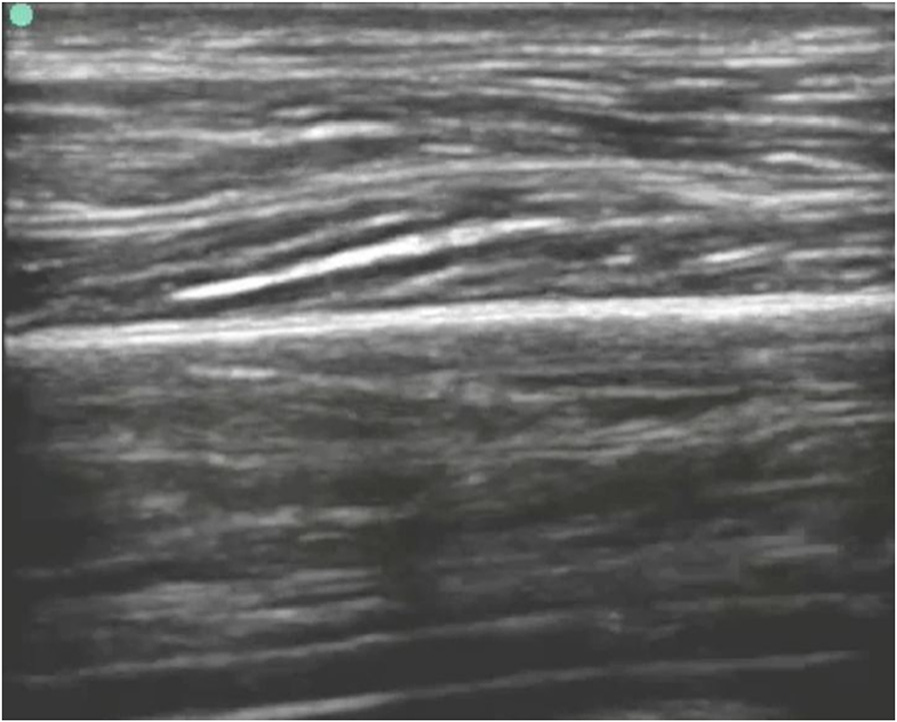
ED bedside ultrasonogram of a normal quadriceps muscle.

**Figure 3:**
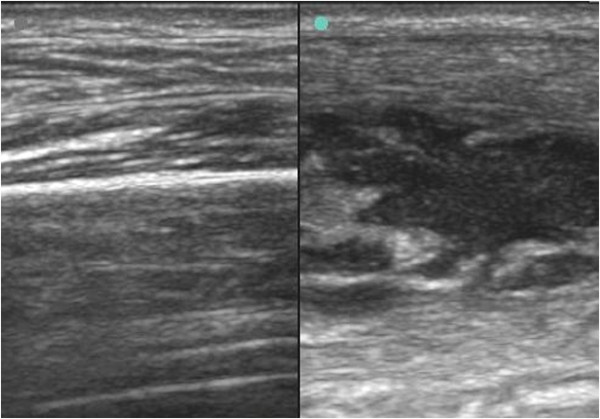
Normal quadriceps muscle (left) and quadriceps intramuscular loculated abscess or pyomyositis (right).

ED bedside dynamic compression ultrasonography was performed on both anterolateral thighs (see Additional files 1, 2, and 3 available as supporting information in the online version of this paper). Examination of the right anterolateral thigh and quadriceps muscle was unremarkable (Figure 2 and Additional file 1). Bedside dynamic compression musculoskeletal ultrasonography examination of the left anterolateral thigh revealed a quadriceps intramuscular abscess with loculated yet movable pus in both the longitudinal axis (Additional file 2) and transverse axis (Additional file 3). The normal right thigh was compared to the left thigh with the intramuscular quadriceps abscess (Figure 3).

Orthopedic and general surgery consultation was obtained in addition to formal radiology ultrasound and computed tomography examinations of the patient’s left thigh and quadriceps muscle, which revealed an intramuscular quadriceps abscess consistent with pyomyositis. The patient was treated with immediate intravenous broad-spectrum antibiotics in the ED. The patient’s condition worsened, and he became septic. He ultimately was admitted to the surgical intensive care unit where he underwent incision and drainage of his intramuscular quadriceps abscess, and eventually, he became hemodynamically stable and was transferred to the general medical floor.

## Discussion

Pyomyositis with intramuscular abscess formation has occurred worldwide especially in Africa in both adults and children; yet in 1971, Levin described the emergence of pyomyositis in North America, with over 600 cases having been reported since [3-7]. Pyomyositis exhibits an overall male predominance, with cases in milder climates affecting primarily adults and the elderly. In addition to other immunocompromised states, such as HIV infection, diabetes, steroid use, and certain hematologic malignancies, trauma has been noted to be an important risk factor, occurring in up to 40% of cases in North America [2]. The emergence of HIV infection in North America has certainly contributed to the increased prevalence of pyomyositis in North America [2]. Primary pyomyositis is due to transient bacteremia as the major cause; yet, secondary pyomyositis can occur from contiguous infectious spread from osteomyelitis or cellulitis [1,2]. While *Staphylococcus aureus* remains the causative organism in the majority of cases, other gram-positive, gram-negative, atypical, parasitic, and viral etiologies have also been reported [2]. Community-associated methicillin resistant *S. aureus* has also been reported to cause intramuscular abscess pyomyositis syndrome [8,9]. Our patient’s recent chemotherapy and his myelodysplasia with lower neutrophil count certainly put him at a much higher risk for developing quadriceps intramuscular abscess and pyomyositis.

Progression of pyomyositis has been described as occurring in three discrete stages [10,11]. Thought to arise via hematogenous spread from a focal source, the early or invasive stage is characterized by nonspecific symptoms, including fever, malaise, and anorexia, and generally lasts approximately 10 days. Examination findings may include minimal swelling and mild overlying tenderness. The second or purulent stage is characterized by progression of local symptoms, including erythema and more pronounced tenderness. Most patients will seek initial medical attention during this phase, when the diagnosis may be mistaken for cellulitis or deep vein thrombosis. The final or late stage involves more clinically overt infection, often meeting the criteria for systemic inflammatory response syndrome or sepsis, with the physical exam progressing to reveal fluctuance of the overlying skin [10,11]. Treatment beyond the first stage of infection involves ultrasound-guided drainage or surgical debridement in addition to antibiotic therapy. Delayed treatment may result in pronounced systemic disease, including metastasis of abscesses to distant anatomical sites, sepsis, and septic shock [10,11].

While magnetic resonance imaging (MRI) is considered the diagnostic gold standard for the diagnosis of pyomyositis, ultrasonography is most useful during the purulent stage of the infection, when it may reveal diffuse muscle hyperechogenicity with or without localized hypoechogenicity and diffuse hyperemia [12-17]. Despite the presence of fluid or pus, abscesses located within the muscle layer may not exhibit the typical sonographic features expected from more superficially located collections. The appearance of muscle isoechogenicity and a solid appearance on a static ultrasound exam may be falsely reassuring for the absence of an intramuscular abscess. A dynamic compression ultrasound exam may be required to aid in the identification of fluid and loculated pus, allowing for the definitive diagnosis of an intramuscular abscess or stage II pyomyositis and for the appropriate management of ultrasound-guided aspiration and drainage or open surgical drainage [13,16]. Direct probe pressure with high-frequency linear array ultrasound probe applied to our patient’s quadriceps muscle produced swirling of the heterogeneous deep-muscle purulent material.

The ED diagnosis of pyomyositis can be challenging especially when patients present with atypical symptoms such as fever with hip, back, or flank pain, especially in patients who are difficult to manage such as intravenous drug users [18-21]. Chern reported on ten patients who presented to the ED with a final diagnosis of psoas intramuscular abscess and pyomyositis, and five or 50% of the patients complained of flank pain, and the triad of fever, flank pain, and a limitation of hip movement, which is specific for psoas muscle abscess, was present in only three or 30% of the patients [18]. Emergency CT or MRI scanning of an ED patient’s psoas muscle would provide the definitive diagnosis of pyomyositis as even a lower frequency ED ultrasound probe may not penetrate to the patient’s retroperitoneal psoas muscle for a definitive diagnosis.

## Conclusions

Bedside point-of-care dynamic compression ultrasonography can assist the emergency or critical care physician in the rapid diagnosis of an intramuscular abscess or pyomyositis. Rapid diagnosis of intramuscular abscess, especially in an immunocompromised patient, can expedite urgent orthopedic surgical consultation.

## Consent

A written informed consent was obtained from the patient for publication of this case report and any accompanying images. A copy of the written consent is available for review by the Editor-in-Chief of this journal.

## Competing interests

The authors declare that they have no competing interests.

## Authors’ contributions

DR and AT drafted and edited the manuscript. Both authors read and approved the final manuscript.

## Authors’ information

DR is the director of Emergency Ultrasonography and Ultrasound Research and AT is an Emergency Department attending physician in the Emergency Medicine Department, Columbia University Medical Center, New York, NY.

## Supplementary Material

Additional file 1 **Emergency department ultrasonography bedside diagnosis of a normal quadriceps muscle.** Video of longitudinal axis evaluation of a normal quadriceps muscle. Click here for file

Additional file 2 **Emergency department ultrasonography bedside diagnosis of dynamic compression of a quadriceps intramuscular loculated abscess.** Video of longitudinal axis evaluation of dynamic compression of a quadriceps intramuscular loculated abscess. Click here for file

Additional file 3 **Emergency department ultrasonography bedside diagnosis of dynamic compression of a quadriceps intramuscular loculated abscess.** Video of transverse axis evaluation of dynamic compression of a quadriceps intramuscular loculated abscess. Click here for file
